# The SDGs and health systems: the last step on the long and unfinished journey to universal health care?

**DOI:** 10.1093/eurpub/ckaa035

**Published:** 2020-05-11

**Authors:** Selina Rajan, Walter Ricciardi, Martin McKee

**Affiliations:** c1 Centre for Global Chronic Conditions, London School of Hygiene & Tropical Medicine, London, UK; c2 Department of Public Health, Università Cattolica del Sacro Cuore, Roma, Italy; c3 European Observatory on Health Systems and Policies, Brussels, Belgium

## Abstract

In 2015, the world’s governments committed, in the Sustainable Development Goals (SDGs), to achieve universal health coverage by 2030, something they will be held accountable for. We examine progress in the WHO European Region using data from several sources. We assess effective coverage using data from the Global Burden of Disease Programme, including access to 9 key interventions for maternal and child health and communicable and non-communicable diseases and mortality from 32 conditions amenable to health care. Progress is mixed; while Finland and Iceland have already achieved the 2030 target already, other countries, including in the Caucasus and Central Asia have not yet, and are unlikely to by 2030. We then examine financial protection, where progress lags in Central and South East Europe and the former Soviet Union, where high out-of-pocket healthcare payments and catastrophic spending are still common. We stress the need to consider inequalities within countries, with the most vulnerable groups, such as Roma or newly arrived migrants (from the Middle East and Africa) often underserved, while their needs are frequently undocumented. To make progress on the SDGs, governments must invest more heavily in health services research and support the infrastructure and capacity required to enable it.

## The quest for universal health care

The United Nations Declaration on Human Rights states that ‘Everyone has the right to a standard of living adequate for the health and well-being of himself and of his family, including food, clothing, housing and medical care…’ (emphasis added). Eighteen years later, in the 1966 International Covenant on Economic, Social and Cultural Rights, many governments went further, recognizing ‘the right of everyone to the enjoyment of the highest attainable standard of physical and mental health’, including ‘the creation of conditions which would assure to all medical service and medical attention in the event of sickness’. A subsequent General Comment introduced the concept of ‘progressive realization’ of the right to health within available resources.^1^

These developments reflect a rights-based perspective, viewing health as a fundamental right attached to being human. More recently, other arguments have been added, such as how health is a pre-requisite for development. This emerged in the 1993 World Development Report ‘Investing in health’^2^ and, subsequently, the Commission on Macroeconomics and Health.^3^

These strands slowly coalesced, with calls for governments to take an active role in ensuring the means to achieve good health and, especially, the adoption of universal health coverage (UHC) through strong and effective health systems. The goals of health systems, set out in the 2000 World Health Report,^4^ are 3-fold, and include improving health, responding to the legitimate expectations of the public and fair financing, with the last of these recognizing the dangers that health systems can lead to catastrophic expenditure by those who fall ill and their families.

Until the mid-2000s, these debates focussed primarily on low- and middle-income countries. However, following a review of the evidence, it was increasingly recognized that similar arguments applied to high-income countries. The evidence showed that it was not only in poor countries (where physical labour in agriculture and extractiveindustries is the predominant form of labour) that poor health was an impediment to economic growth. It was also important in rich countries, reducing labour supply, through premature withdrawal from the labour force or working fewer hours, decreasing productivity and lowering the propensity of individuals to invest in their own education or to save for the future.^5^

Finally, in 2015, the governments of the world committed, in the Sustainable Development Goals (SDGs),^6^ to achieve UHC. Health had featured in the earlier Millennium Development Goals, but in a minimalist way, limited to pursuing reductions of deaths of mothers and children and incidence of a small number of infectious diseases. The SDGs are much more ambitious. And while the challenges are greatest in the poorest countries, it is a call to action in all.

The European Region is, overall, the most developed of WHO’s regions. Yet it is extremely diverse, ranging from the Nordic countries with their long-established and highly developed health and social systems to some of the countries in Central Asia that continue to struggle to provide basic health care for large sections of their populations. Moreover, even in the wealthiest countries, there are groups in the population that are excluded, lacking access to care. In this article, we take stock of what the European Region has achieved so far and what it has still to do to hit this goal.

## Ensuring healthy lives and promote well-being for all at all ages

SDG 3 is to ensure healthy lives and promote well-being for all at all ages. This has been broken down into a series of targets, with one, Target 3.8, to ‘achieve UHC, including financial risk protection, access to quality essential healthcare services and access to safe, effective, quality and affordable essential medicines and vaccines for all’. It is to be measured against two indicators, 3.8.1, defined as ‘Coverage of essential health services [defined as the average coverage of essential services based on tracer interventions that include reproductive, maternal, newborn and child health (RMNCH), infectious diseases, non-communicable diseases and service capacity and access, among the general and the most disadvantaged population] by 2030’ and 3.8.2, defined as ‘Proportion of population with large household expenditures on health as a share of total household expenditure or income’. The challenge is how to operationalize these.

Indicator 3.8.1 refers to tracer interventions rather than the outcome measures that were used in the MDGs, including infant, under-5 and maternal mortality. The MDGs were criticized because they seemed to be driven by what was already measured in low- and middle-income countries, creating a framework that was less relevant to the European Region,^7^ so this SDG seems an improvement, but the question is where the necessary data will come from?

An inter-agency task force, established by the WHO and World Bank, has agreed a process to develop indicators of UHC.^8^ The task force has established four guiding principles for developing a composite index of health service coverage. First, the components should be measures of effective service coverage. Second, they should cover different types of services, including prevention, treatment, rehabilitation and palliation, explicitly including interventions delivered in other sectors. Third, the index should cover all of the main areas of health set out in the target. Fourth, the index should be disaggregated by key dimensions of inequality. This was an extremely challenging task. Thus, the indicators had to be relevant, reflecting the burden of disease and the existence of cost-effective interventions. Second, it should be feasible, a particular problem given the limited amount of data in many countries. Third, it should be conceptually sound, with a clear target and a definition that captures effective coverage. And finally it should be easy to communicate.

The resulting index included 16 tracers, although there were insufficient data to calculate two of them. Communicable diseases and maternal and child health dominated, reflecting what is measured in low- and middle-income countries. Non-communicable diseases were captured by hypertension control, mean fasting glucose and smoking rates, as a measure of tobacco control. Cancer detection, proxied by coverage of cervical cancer screening, was also included but it was one of the two indicators for which data were unavailable.

This index, while a major advance on what has gone before, has a number of limitations, some of which are especially relevant to Europe. The report^8^ (page 18) identifies a need for ‘Increasing relevance to higher income countries. Many high-income countries are approaching 100% coverage for tracer indicators in the RMNCH and service capacity and access categories. Other tracer indicators, or a hybrid method that incorporates avoidable mortality, should be assessed’.

**Figure 1:**
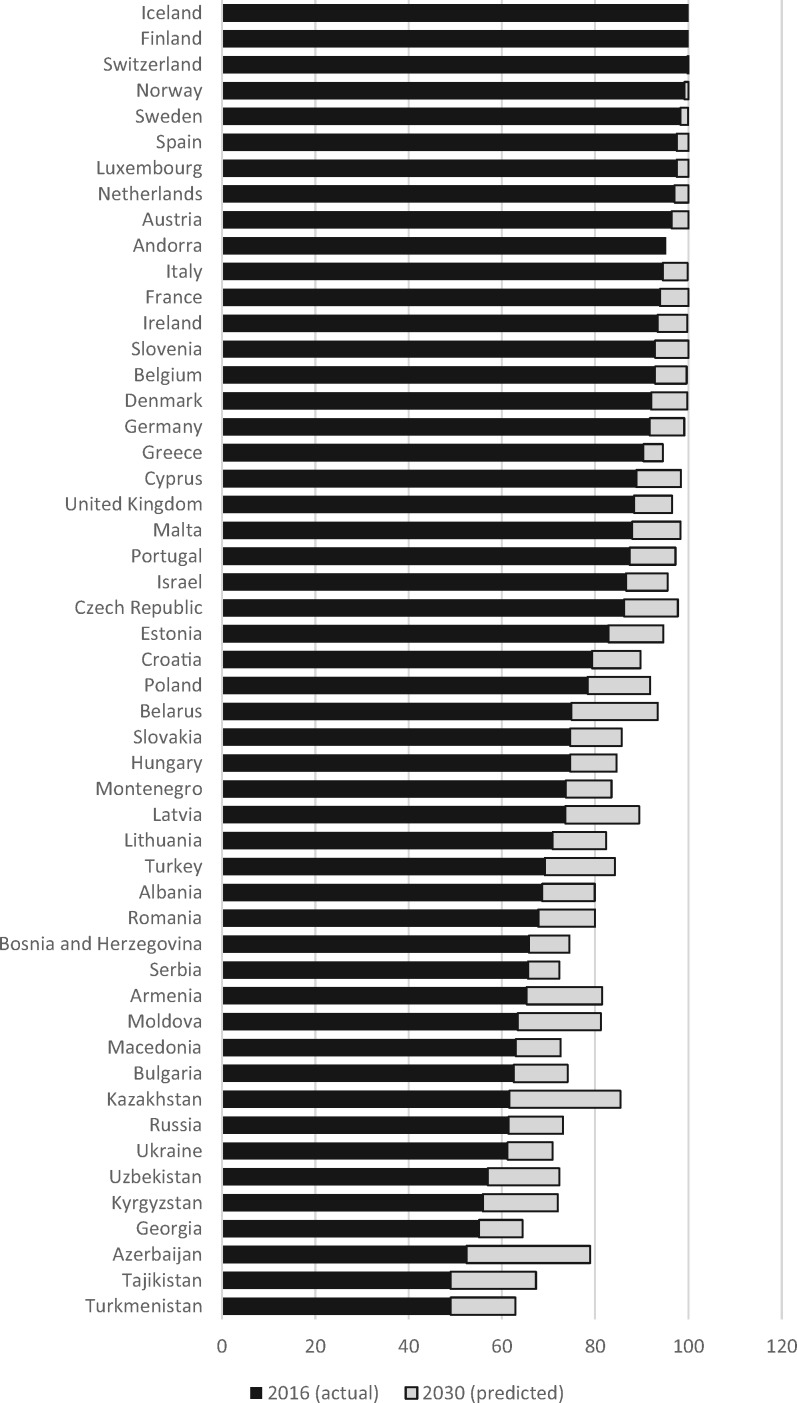
Performance of countries in the WHO European Region on the Global Burden of Disease measure of SDG indicator 3.8.1 (effective coverage) *Note:* For comparison, 2019 values for selected countries are USA, 90.3; Brazil, 63.8; China, 80.5; and Nigeria, 25.9. *Source:*https://vizhub.healthdata.org/sdg/.

This challenge has been taken up by the Global Burden of Disease (GBD) programme. They identified 52 SDG indicators that could be considered health-related.^9^ These went beyond those associated with SDG 3. Thus, SDG 1, to end poverty, SDG 2, to end hunger, and several others have a clear health dimension. Of the 52 that were so identified, they identified 41 that could be measured using data already collected in the GBD. For example, for target 1.5, to increase the resilience of poor and vulnerable people to external shocks, they used the death rate from a group of causes categorized as forces of nature. For target 6.2, to eliminate intimate partner violence against women and girls, they used a measure of the prevalence of this experience. For each indicator, they collated or estimated data from 1990 to 2016 and forecast future progress to 2030, based on a weighted analysis of historic trends. They then developed a scale from 0 to 100, with 0 as the 2.5th percentile and 100 as the 97.5th percentile of 1000 draws calculated from 1990 to 2030, before calculating the geometric mean. The indicator used to track progress to Goal 3.8 is defined as ‘Coverage of essential health services, as defined by a UHC service coverage index based on 9 tracer interventions and risk-standardized death rates or mortality-to-incidence ratios from 32 causes amenable to healthcare’. The tracer conditions are largely the same as those used by the WHO. The difference is the inclusion of the causes of death amenable to healthcare. These are taken from another major component of the GBD, the development of the Health Access and Quality Index, which captures mortality from conditions in which death should not occur in the presence of timely and effective health care.^10^ This to some extent overcomes the problem with the WHO index, which fails to discriminate among high-income countries. Figure 1 shows the estimates for 2019 and the forecast for 2030 for the countries of the European region of the WHO. On this measure, Finland and Iceland have already achieved the target for 2030, with some others very close behind. Several European Union member states still have some way to go, and on current trends will fail to achieve the target by 2030. However, the greatest problems are in the eastern part of the region, and in particular in the Caucasus and Central Asia, where even with the forecast improvements, they will still be far short of the target by 2030.

Notwithstanding the progress that has been made in defining these indicators, neither address the crucial words in the Goal ‘among the general and the most disadvantaged population’. Here, an important question is who are the most disadvantaged group in a population. There is no simple answer as the characteristics of groups that are excluded depend on features of the particular health system, of the wider society in which it is embedded, and its history, geography and contemporary politics. Thus, they may include indigenous populations, such as Sami in Lapland, those defined by ethnicity, which in Europe includes both Roma and more recent arrivals, socioeconomic characteristics, such as poverty, insurance status and citizenship or migration status.

This challenge was addressed in a recent report by the European Commission’s Expert Panel on Investing in Health, which was asked to provide an opinion on ways of benchmarking unmet need for healthcare within the European Union, as a means to measure progress on the European Pillar of Social Rights. The report recommended that unmet need be measured using responses to a question in the EU Survey of Income and Living Conditions (EU-SILC), conducted in all EU Member States, which asks respondents whether they have experienced a time in the previous 12 months in which, in their opinion, they needed medical care, but did not receive it. Subsequent questions and identification of whether this was on grounds of affordability, availability and user experience. These data can be disaggregated by a range of socioeconomic and demographic parameters. Thus, the report shows inequalities in levels of unmet need according to gender, age, poverty, education and employment. However, the report notes that, beyond these important divisions within the population, there are often groups that suffer extreme disadvantage. These include undocumented migrants, particular ethnic and religious minorities and homeless people. Importantly, those most vulnerable often differ among countries, making comparisons difficult. This may be because of differences in their share of the population, e.g. because of the very different levels of migration from different parts of the world to individual European countries, or because of cultural and historical factors, or the inclusiveness of social policies. Consequently, the report proposed that each country should identify those groups that are especially vulnerable and conduct a detailed quantitative and qualitative assessment of the extent and nature of the unmet need the experience, and the barriers that must be overcome.

Undertaking such assessments is not always easy. Vulnerable populations may, rightly, fear contact with authorities. Skilled researchers, with a detailed understanding of specific cultural attributes, are often in short supply. As a result, much of this research has, so far, been conducted in a very small number of countries, almost entirely in Western Europe.

Europe’s largest minority comprises the Roma population, concentrated in Central and Eastern Europe, but with significant numbers elsewhere, such as Spain. There is extensive evidence that they have higher levels of unmet need for healthcare in many countries and, unfortunately, this is often the result of institutionalized discrimination, manifest as culturally appropriate care, lack of facilities in Roma communities and administrative barriers, such as making it difficult to acquire identification papers.^11^ While some progress was made during the recently completed Decade of Roma Inclusion, there is still much to be done.

For other groups, progress that was being made is now going into reverse. Migration from outside Europe is fuelled by conflict, political oppression, impoverishment and, increasingly, the effects of climate change. Countries that once tolerated, or even welcomed migration are closing their borders. Those who manage to reach Europe face increasing barriers to obtaining healthcare. The UK for example has created a ‘hostile environment’, whereby public services make it as difficult as possible for undocumented migrants to obtain support.^12^ These have, however, blocked people who are entitled to care from receiving it, especially those who arrived from then British colonies in the Caribbean as children, but are unable to comply with the extremely complex documentary requirements to prove their British citizenship. In contrast, in 2018 a new Spanish government reversed restrictions imposed by its predecessor.^13^

Finally, we turn to indicator 3.8.2, capturing large household expenditures on health. People in some countries in Central and South East Europe and the former Soviet Union have long faced high out-of-pocket payments for health care, with frequent catastrophic payments. Reasons include gaps in coverage, with many people who have irregular or undocumented status being excluded, frequent formal payments, especially for medicines, and widespread informal payments.^14^

Things are better in Western Europe but, even here, there are no grounds for complacency. Recent work by the Barcelona Office of WHO Europe^15^ goes beyond the traditional measure of catastrophic spending to include ‘impoverishing spending’, where a household is taken below the poverty line by spending on health, and ‘further impoverishment’, where it is already below the line but its resources are reduced further. This is arguably a more appropriate measure for high- and upper-middle-income countries. Using this measure, between 1% and 5% of households in some EU member states face impoverishment on account of health expenditure each year, with co-payments playing an important role.

## Summary

The SDGs are a major advance on the MDGs, in several ways, not least of which is the scope of what is covered. However, for Europe, perhaps the most important change is that they apply to countries at all levels of development. All governments, across the entire European Region, have committed to achieving goals in many sectors that will improve the health of all of their populations.

So how are they doing? Before answering this question, it is necessary to reflect on the limitations of the data. While the GBD data are the most comprehensive, resulting from a massive effort to collect, evaluate and combine information from numerous sources, for some countries it is only possible to use estimates. For many of the indicators, there are no routinely collected data and, where surveys are used they may be undertaken irregularly or use questions and sampling methods that are not fully compatible. In particular, they often exclude, by design or unintentionally, certain marginalized groups, such as undocumented migrants and people who are homeless or in institutions.

While recognizing the issues with the data sources, this brief review paints a mixed picture. Some European countries have already achieved very high levels of coverage of essential health services. But others, including some very wealthy ones, still have some way to go. Looking across the entire region, there is great diversity. The challenges in the Central Asian republics and the Caucasus are especially great.

Yet, even in those countries that are, overall, doing well, there are a number of vulnerable groups who risk being left behind. Often, we know very little about these groups and, especially, their health needs and the challenges they face in obtaining care. They are, in many ways, invisible.

The situation is less reassuring when it comes to financial protection. The work of the WHO Barcelona office has revealed that many people, even in some of the richest countries in Western Europe, are facing surprisingly high levels of out-of-pocket payments. Clearly, there is much that needs to be done to strengthen the financial protection offered by health systems.

There are, however, some reasons for hope, despite the many current geopolitical challenges, in particular the ascendency of populist politicians in some countries pursuing policies that are contrary to their commitments in the SDGs. Many governments have recognized the role of health systems in achieving two related policy objectives.^16^ One is sustained economic growth, with a health workforce contributing through greater labour force participation and productivity.^5^ The other is social cohesion, as it becomes apparent that worsening health feeds populist sentiment.^17^ These concepts came together at a WHO conference in Tallinn in 2018, with the theme Include, Invest, Innovate.^18^

So what needs to be done to achieve the targets directed at health systems? The SDGs are important for many reasons, but one is that they provide a framework for accountability. It cannot be said often enough that governments have committed to achieve them. They must be held to account. This can only happen, however, if the evidence on progress that is being made, or in some cases not being made, is visible. This supplement seeks to do this, but inevitably, it will only reach a relatively small audience. It is important that those engaged in health and related policies, either as policymakers, researchers or practitioners, take this information, much of which is easily obtainable, e.g. from the GBD data visualizer (http://www.healthdata.org/gbd/data-visualizations) and place it in front of the public, politicians and the media. If news bulletins can give prominence to the latest data on economic growth, surely we should expect them to do the same with data on the health of the population.

Yet it is not enough to show that there is a problem. It is also necessary to offer solutions. This will require a substantial investment in health services research in all parts of Europe, supported by appropriate infrastructure, including a significant enhancement of data systems. While the EU-SILC data do provide some basic comparative information within the EU, they have considerable limitations,^19^ and there is nothing comparable in the rest of the European region. There is also a need to strengthen research capacity. In recent years, health services research has been less prominent in the EU research agenda, with its focus on industrial innovation. There are some signs that this is changing, and projects such as TO-REACH are pointing to what needs to be done.^20^

The SDGs are a tremendous opportunity for those who are seeking to improve the health of the people of Europe, and of the world, and it is important that we do not let this opportunity pass us by.

*Conflicts of interest*: We declare no conflicts of interest.
